# Facile Synthesis of Ternary Alloy of CdSe_1-*x*_S*_x_* Quantum Dots with Tunable Absorption and Emission of Visible Light

**DOI:** 10.3390/nano8120979

**Published:** 2018-11-27

**Authors:** S. Wageh, Ahmed Al-Ghamdi, Asim Jilani, Javed Iqbal

**Affiliations:** 1 Department of Physics, Faculty of Science, King Abdulaziz University, Jeddah 21589, Saudi Arabia; aghamdi90@hotmail.com; 2 Physics and Engineering Mathematics Department, Faculty of Electronic Engineering, Menoufia University, Menouf 32952, Egypt; 3 Center of Nanotechnology, King Abdulaziz University, Jeddah 21589, Saudi Arabia; asim.jilane@gmail.com (A.J.); iqbaljavedch@gmail.com (J.I.)

**Keywords:** quantum dots, ternary alloy, optical absorption, luminescence, color analysis

## Abstract

The synthesis of alloyed semiconductor quantum dots has produced structures that have distinct properties in comparison with both their bulk counterparts and their parent binary semiconductor quantum dots. In this work, the quantum confined structures of a ternary alloy of CdSe_1−*x*_S*_x_* were synthesized by one-pot synthesis method in an aqueous medium at a low temperature and capped with 3-mercaptopropoionic acid. Structures of the synthesized quantum dots were investigated by energy dispersive X-ray, X-ray diffraction, X-ray photoelectron spectroscopy, and high-resolution transmission electron microscopy. The obtained quantum dots had modified cubic structures as proven by X-ray diffraction and selected area electron diffraction. The optical properties of the synthesized quantum dots were characterized by optical absorption, photoluminescence, and color analysis. Optical absorption investigation revealed a widening of the band gap of CdSe_1−*x*_S*_x_* with increasing S content. This widening increased for the samples suspended in water relative to the samples measured in powder form due to the difference in the environment of the two cases. The size determined from the optical absorption measurements was found to be compatible with the sizes obtained from the X-ray diffraction with the value of bowing parameter around 1, which indicated a graded diffusion of sulfur. It was also ascertained that the emission of different compositions covered the most visible range with a small full width at half maximum. The *x* and *y* values of the chromaticity coordinates decreased with increasing sulfur content of up to 15%, while the *z* value increased.

## 1. Introduction

The quantum confined structures of semiconductors have been extensively investigated in the last few decades due to their outstanding physical and chemical properties [1,2]. In these structures, the quantum confinement effects control the properties of the materials with a dramatic change in their properties by varying the size of the nanocrystallite [1,2]. This behavior awards the semiconductor nanostructures and corroborates different applications such as photovoltaics [3,4,5], photocatalysis [6,7,8], lasers [9], transistors [10], biological labels [11], and light emitting devices [12,13]. The tunable physical and optical properties of semiconductor nanostructures can be achieved by two ways: the first by changing the size and dimension of the nanocrystals in the range below the exciton Bohr diameter; and the second is the variation of the optical and physical properties of the nanocrystallite by regulating the constituent stoichiometries of the alloy compounds. These two ways produce a compound that can possess outstanding properties to those of the bulk compounds. In alloying compounds of two binary semiconductors, it is possible to vary the optical and physical properties by changing the constituent composition of the nanostructure. This can be carried out by forming ternary semiconductors that are composed of two binary compounds that possess different energy gaps. In this way, an increase of the ternary semiconductor band gap can be achieved by increasing the content of the wider band gap compound. In contrast, the decrease of the ternary semiconductor band gap can be achieved by increasing the content of the narrower band gap binary compound. According to the diffusion of the third element in the original pair of semiconductors, ternary nanocrystal alloys can be divided into two classes: the first structure is uniform, which are called homogenous structures; and the second class where the composition and structures change radically. These two types of structures have different arrangements in comparison with the core shell structure. The core shell structure consists of a narrower band gap semiconductor in the core shielded by a thin layer of wider band gap compound. The growth of the ternary semiconductor compound can be carried out by combining two binary compounds that possess common anions or common cations. Rare works have been published on ternary alloy nanostructures that have common anions like as CdHgTe [14], CdZnSe [15], CdZnS [16], and CdZnTe [17]. Additionally, some scarce works have been reported on common cation ternary alloy nanostructures such as CdTeSe [18,19], CdSTe [20], and ZnSTe [21]. Among some common cation ternary compounds, CdSSe is a very good candidate for the next generation of biomedical and optoelectronic applications. The CdSSe alloy has become a focal point for researchers due to its captivating and alterable optical and electrical properties. CdSSe carries the properties of both CdSe and CdS where the band gap can be changed from visible (∼2.42 eV for CdS) to near IR (∼1.73 eV for CdSe) [22]. The composition of elements in the alloy plays a pivotal role in tuning the band gap. Its fantastic optical properties (fast response times, large nonlinear susceptibilities, and good photoconduction) make it a suitable candidate for a wide range of potential applications in next generation optoelectronic devices [23,24] and biomedical science, such as drug delivery, biosensors, in vivo imaging, and fluorescent labelling [25,26]. As a matter of fact, a deeper understanding of CdSSe quantum dots with a highly controllable facile synthesis protocol could meet the demand of future applications.

Accordingly, the preparation of CdSSe with tunable optical properties is considered as an essential demand. Ternary CdSSe quantum confined structures have been prepared by the precipitation method in dimethyl sulfoxide DMSO with sizes in the range from 5 to 10 nm [27]. Two years later, in 2003, CdSSe ternary nanostructures with a size nearly equal to 5 nm were prepared by the rapid hot injection method through the injection of chalcogenide sources into Cd precursors [28]. In 2005, the CdSe–CdS alloy was prepared by microwave irradiation through the preparation of a selenium source in an oxygen free environment by using NaBH_4_ and selenium powder [29]. In 2006, CdSe_1−_*_x_*S*_x_* alloy nanostructures were prepared by the pyrolytic synthesis method based on a hot injection method [30]. Three years later, CdSeS nanocrystal alloys were prepared at 240 °C with myristic acid (MA), 2,2′-dithiobisbenzothiazole (MBTS), and 1-octadecene [31]. One year later, CdS*_x_*Se_1−*x*_ alloy nanostructures were prepared in oleic acid (OA, tech. 90%) and 1-octadecene at 240 °C [32]. In 2013, CdS*_x_*Se_1−*x*_ ternary alloy nanostructures were prepared by modifying the two phase method using cadmium myristate, oleic acid or tri-*n*-octylphosphine oxide (TOPO) in toluene [33]. All of these methods of preparation employed high temperature using tri-*n*-octylphosphine oxide (TOPO)/tri-*n*-octylphosphine or other long chain organic materials. The use of such materials requires rigorous conditions for preparation. Accordingly, finding a new synthetic method to produce ternary alloys is still a challenge.

The one-pot synthetic method can be considered as a useful way to produce a large number of nanostructures and save time along with reducing the costs of the preparation of nanocrystal semiconductors. Preparation of binary, ternary, doped CdSe, and core-shell structures at high temperature were done through the one-pot synthetic method. CdS and CdSe binary compound nanocrystals were prepared in hot organic solvents under high temperatures in the range between 200 and 300 °C [34,35,36,37,38]. Additionally, CdSe nanocrystals were synthesized via the one-pot method using liquid paraffin as a solvent at high temperature (180–260 °C) [39]. On the other hand, CdSe/CdS core-shell nanoparticles were prepared by the one-pot synthesis method in an organic solvent at a high temperature from 240 to 300 °C in a free oxygen atmosphere [40,41,42]. The CdSe/ZnO core-shell was prepared by a one-pot protocol at 260 °C [43]. CdSe/CdS/ZnS core/multishell nanocrystals were prepared by the one-pot method at a high temperature of 300 °C under a free oxygen atmosphere environment [44]. Additionally, CdSe/CdS/CdZnS core/multishell nanocrystals were prepared at a relatively low temperature between 100 and 130 °C inside a microwave digestion furnace [45]. CdSe semiconductors doped with Au and Eu were prepared by the one-pot synthesis method in an organic solvent at a temperature ranging from 120 to 280 °C [46,47]. In addition, a Cu-doped CdSeTe-alloyed semiconductor was prepared via a non-injection one-pot approach in an organic solvent at high temperature [48]. CdSe doped with Mn encapsulated in carbon was prepared by the one-pot method under autogenic pressure at an elevated temperature [49]. The one-pot preparation method was also applied to prepare alloy CdTeSe nanocrystals with a magic-size in a high-temperature organic solvent at 180 °C [50].

To the best of our knowledge, the preparation of a CdSSe ternary alloy using the one-pot method at low temperature without the need of a free oxygen atmosphere and with a combination of cadmium acetate dehydrate, sodium selenite, 3-mercaptopropionic acid (3-MPA), and sodium sulfide in the presence of reducing agent has yet to be reported.

In this work, the one-pot synthesis method of CdSe_1−*x*_S*_x_* quantum dots with *x* = 0, 0.05, 0.1, 0.15, and 0.25 at low temperature is reported. By changing the S content from 0 to 25%, the emission could be tuned from green to blue.

## 2. Experimental

### 2.1. Materials

The chemicals used in this study were all of an analytical grade and included the following: cadmium acetate dihydrate (Cd(CH_3_COO)_2_·2H_2_O, Sigma Aldrich, Chemie GmbH, Buchs, Switzerland), sodium selenite (Na_2_SeO_3_) (Panreac Quimica SAU, Barcelona, Spain), 3-mercaptopropionic acid (3-MPA) ((C_3_H_6_O_2_S), (Fluka Chemie GmbH, Buchs, Switzerland)), sodium borohydride (NaBH_4_, Spectrosol, BOH Chemicals, Ltd. Poole, UK), sodium sulfide (Na_2_S·9H_2_O, Swiss medicare, West Yorkshire, UK); sodium hydroxide (NaOH, BOH Chemicals Ltd. Poole, UK); acetone, ethanol, isopropyl alcohol (Sigma-Aldrich Co., St. Louis, MO, USA); and ultrapure water.

### 2.2. Preparation Method

In the current study, ternary CdSe_1−*x*_S*_x_* quantum dots were synthesized by the colloidal aqueous approach in a one-pot synthesis. This process depends on the reaction of cadmium ions (or complexes) with chalcogenide precursors in the presence of organic stabilizing/capping agent molecules (in this study organic bifunctional molecule), which contain a soft acidic group (thiol) and hydrophilic group (carboxyl). The one-pot synthesis of alloyed MPA-capped CdSe_1−*x*_S*_x_* quantum dots was conducted using a simultaneous injection of Na_2_SeO_3_ and Na_2_S·9H_2_O into the Cd precursor aqueous solution. The variation in the composition could be achieved by changing the relative amount of the starting precursors of the different anions. The main steps can be summarized as follows: dissolve 5.63 mmol cadmium acetate dihydrate in ultrapure water with continuous stirring, followed by the addition of 10.33 mmol of 3-mercaptopropionic acid (3-MPA) under constant stirring in an ambient atmosphere in a three-neck flask. The solution became turbid with a white color after adding 3-MPA. The pH value was adjusted to 10.2 using a sodium hydroxide (NaOH) solution to have a clear and transparent mixture. Next, 5.65 mmol of a selenium source of sodium selenite (Na_2_SeO_3_) was injected into the mixture for the preparation of binary CdSe. Finally, 3.32 mmol of sodium borohydride was added into the solution mixture. For ternary CdSe_1−*x*_S*_x_* quantum dots, Na_2_SeO_3_ and Na_2_S·9H_2_O with different ratios were simultaneously injected into the mixture of cadmium salts along with capping molecules. The temperature of the mixture gradually increased to 100 °C and the solution kept refluxing for 200 min. The reaction conditions were kept the same for all samples (molar ratio of starting precursors, pH, temperature, time) except for the difference in chalcogenide source.

Binary CdSe and ternary CdSe_1-*x*_S*_x_* quantum dots prepared with the presence of 3-mercaptopropionic acid were obtained with the help of a reducing agent of sodium borohydride to proceed with the reaction of selenium ions from Na_2_SeO_3_, which may be considered as the following:

(1) $$4{\mathrm{Se}O}_{3}^{2-}+3{BH}_{4}^{-}\;\to \;4{\mathrm{Se}}^{2-}+3{BO}_{2}^{-}+6H_{2}O$$

(2) $${n(\mathrm{Cd}}^{2+}{\;-\;3\mathrm{MPA})\;+\;nxS}^{2-}{\;+\;n(1-x)\mathrm{Se}}^{2-}\;\to {\;(\mathrm{CdSe}}_{1-x}S_{x}{)}_{n}{\left(3\mathrm{MPA}\right)}_{m}+n-m\left(3\mathrm{MPA}\right)$$

The synthesized powders of binary CdSe and ternary alloy CdSe_1−*x*_S_*x*_ quantum dots were obtained from the crude solution by adding isopropyl alcohol drop-wise until some flocculation appeared. Afterwards, the nanoparticles were collected by centrifugation and were thoroughly washed with water, ethanol and, finally, with acetone several times. The precipitated particles were dried in a desiccator for several days. The first precipitated powder from each sample was used for further investigation and characterization. Different contents of sulfur were prepared with *x* = 0.0 0.05, 0.1, 0.15, and 0.25 and hereafter called S0, S05, S10, S15, and S25, respectively.

## 3. Characterization

The surface characterizations were performed by field emission scanning electron microscopy (FESEM-JSM7600F JEOL, Tokyo, Japan) and the compositions were measured by energy dispersive spectroscopy using a FESEM attached with an Oxford EDS system. Magnification and voltages are mentioned at the bottom of every image. The X-ray diffraction (XRD) technique was used to investigate the crystal structures of the prepared quantum confined structures. The analysis was performed using an Ultima-IV Rigaku XRD system (Tokyo, Japan) with Cu–Kα radiation (λ = 1.54060 nm). The samples were measured at room temperature using 40 kV and 40 mA.

The elemental surface compositions of the prepared binary and ternary quantum dots were investigated by X-ray photoelectron spectroscopy (XPS, ULVAC-PHI. Inc., Chigasaki, Kanagawa Prefecture, Japan). The XPS spectra were obtained by a scanning XPS microprobe (PHI 5000 Versa Probe II, ULVAC-PHI. Inc., Chigasaki, Kanagawa Prefecture, Japan). The X-ray source was Al–Kα (*hν* = 1486.6 eV) with a spot size of 200 µm and power of 50 W. The spectra of XPS were obtained with an analyzer pass energy of 187.85 eV, with an energy step of 0.1 eV along with a take-off angle of 45° with respect to the surface plane of the film. The fitting of XPS data was obtained by a computer program, called Multipack Software (VERSION 9, ULVAC-PHI, Inc. ULVAC-PHI. Inc., Chigasaki, Kanagawa Prefecture, Japan) to allow for the fitting of Gaussian-Lorentzian line shapes and the effects of spin-orbit splitting to analyze the elemental concentration, estimation of chemical state percentage, and to interpret the XPS versa probe II data. UV–Vis optical absorption was measured in the spectral range of 200–800 nm by a Jasco V770 UV–Vis spectrophotometer (Tokyo, Japan) attached to a 60 mm integrating sphere. A Jasco spectrofluorometer (FP8500, Tokyo, Japan) was used to measure the emission and color analysis of the prepared nanostructures. Luminescence and color analysis were measured by a Jasco spectrofluorometer (FP8500). High-resolution transmission electron microscopy images were performed for the prepared ternary quantum dot using Tecani G2 F20 Super Twin TEM Microscope with a LaB6 emitter (FEI Company, Thermo Fisher Scientific, Eindhoven, The Netherlands). An Eagle 2K HR 200 kV CCD camera was used to collect the images and selected area diffraction (SAD) patterns. For the HRTEM analysis, a small amount of quantum dots were dispersed in ethanol using an ultrasonic apparatus. The dispersed quantum dots were placed on the copper micro-grid by putting a couple of drops of ethanol solution with a micropipette. The grid was then dried in a controlled plasma cleaning chamber for 10 s and transferred to the analysis chamber in the HRTEM.

## 4. Results and Discussion

### 4.1. Energy Dispersive X-ray Spectroscopy

**Figure 1:**
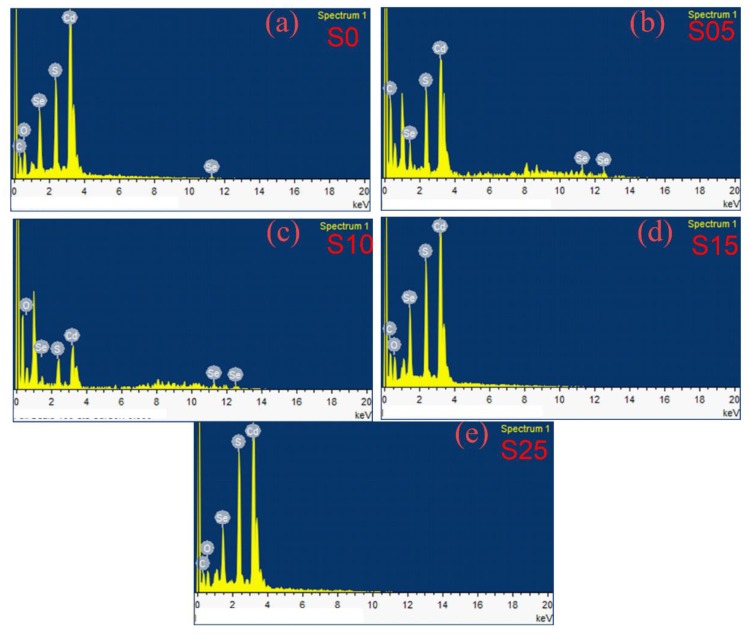
EDX spectra for the CdSe_1−*x*_S*_x_* nanostructures with different content of sulfur. (**a**) *x* = 0.0 (S0 sample); (**b**) *x* = 0.05 (S05 sample); (**c**) *x* = 0.1 (S10 sample); (**d**) *x* = 0.15 (S15 sample); (**e**) *x* = 0.25 (S25 sample).

Figure 1 exhibits the EDX spectra of the binary CdSe and ternary alloy with different contents of sulfur. The appearance of the cadmium and selenium peaks in the EDX spectra of binary CdSe confirmed the preparation of a cadmium selenide core. Additionally, the existence of C, O, and S elements is characteristic of the organic capping shell on the surface of the quantum dots. The calculated S/Se + S atomic ratios are listed in Table 1. The percentage of sulfur that appeared for the binary CdSe was capped by the capping molecule coordinated to the surface of quantum dots and some of the graded diffusion of sulfur may have occurred from the decomposition of 3-MPA. For the ternary alloy, the percentage of S content increased gradually with an increasing source of sulfur in the precursors. In addition, the amount of S content calculated from the EDX was larger than that applied during the preparation, which was due to the S from thiolate at the surface of the quantum dots.

### 4.2. X-ray Diffraction

**Figure 2:**
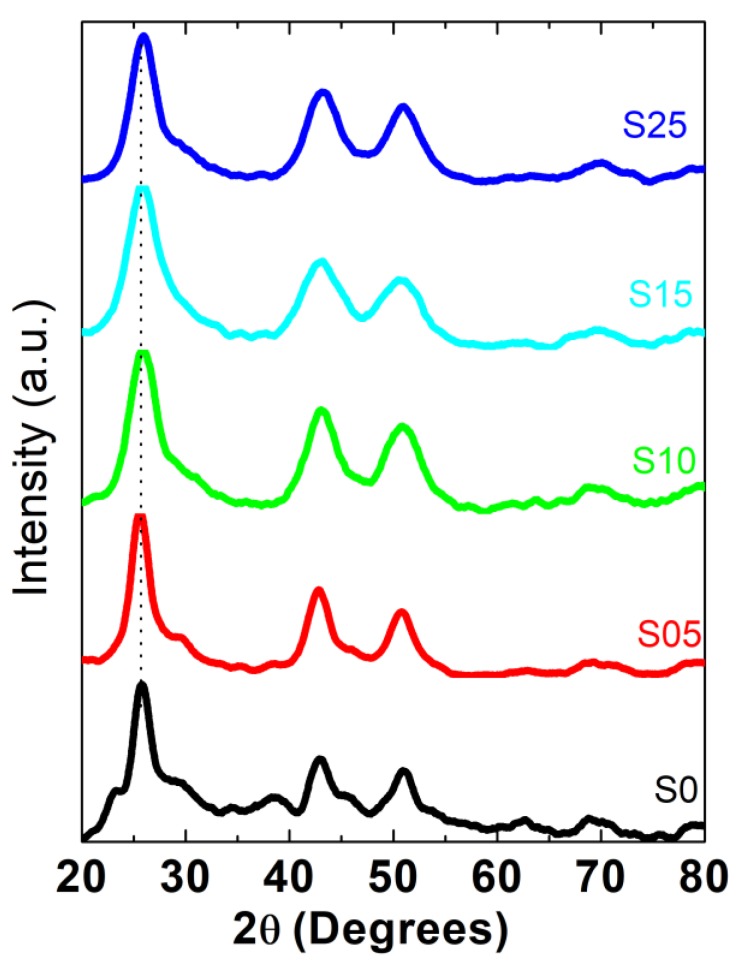
X-ray diffraction patterns of the CdSSe alloy crystal with different percentages of sulfur.

The X-ray diffraction (XRD) technique was used to investigate the type of crystallinity and to determine the sizes of the nanocrystallites. The XRD diffraction patterns measured for the precipitated powdered fractions of CdSe and CdSSe nanocrystals synthesized with different compositions are shown in Figure 2. The diffraction lines of binary CdSe nanocrystallites belonged to the modified cubic structure phase, which is also from the predominant phases of bulk CdSe. Similar results were obtained for the CdS, CdTe, and CdSe nanostructures capped with thiol compounds [51,52].

**Figure 3:**
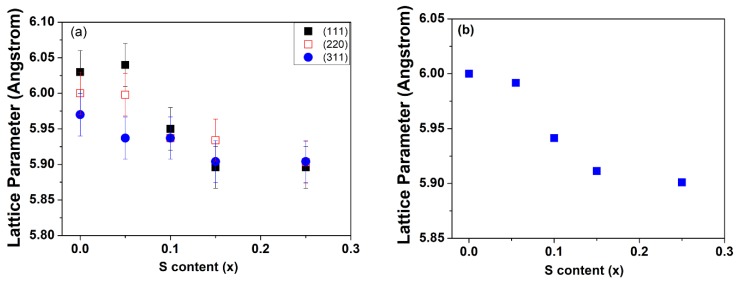
(**a**) Lattice parameters calculated based on a position of different planes. (**b**) The mean lattice parameters calculated based on a position of different planes using ANOVA methods.

The X-ray diffraction patterns of the CdSSe alloy crystal with different percentages of S showed a polycrystalline nature and three predominant diffraction lines appeared for all crystallites with different percentages of S. The appeared diffraction lines of the CdSSe alloy lay at a position between a zinc blend of CdSe and a cubic zinc blend of CdS. In addition, the main diffraction lines indexed (111) shifted a little to the lower angle for the smallest S content while for a high content of S, the (111) position peak gradually shifted to a higher angle with an increasing S percentage. The two diffraction planes (220) and (311) of CdSe presented different behaviors with increasing S percentage. This behavior is similar to that of pseudobinary alloys that have the position of diffraction peaks lying in between the relative two binary compounds [30]. Lattice parameters based on a position of different planes were calculated for the different content of S and are depicted in Figure 3. Different values of lattice parameters were obtained from different planes and the variation behavior of lattice parameters with a changing composition was found to be different for different planes. These results were not like the role of typical semiconductor alloy structures where the variation of lattice parameters follows Végard’s law. The obtained behavior was similar to the compositionally graded nanostructure with increasing sulfur content. The overall general trend of lattice parameters that were calculated from different lattice planes decreased with increasing sulfur content. This result precludes the phase separation or separated nucleation of CdS nanocrystals and represents the alloy growth.

The appearance of broadening and increased breadth of the diffraction peaks arose from the small size of the nanocrystallites. Scherrer describes the breadth of a diffraction peak as being the full width at half maximum of the peak and related it with the size through:

(3) $$D\;=\;\frac{0.9\;\lambda }{\beta \cdot \cos\theta }$$

where D is the diameter of the nanocrystallites; λ is the X-ray radiation wavelength; θ is the diffraction angle; and β is the full width at half maximum (FWHM) of the diffraction peak calculated in radians. The nanocrystallite sizes of the alloyed structure along with binary CdSe were calculated from X-ray and are listed in Table 1. Obviously, the sizes of the alloyed structures prepared with different percentages of S were smaller than the binary CdSe quantum dots. This decrease in the size of the alloyed structure despite all samples being prepared with typical conditions can be attributed to the decrease of ionic strength of the reactive species during the preparation of the alloyed structure.

### 4.3. Transmission Electron Microscopy

**Figure 4:**
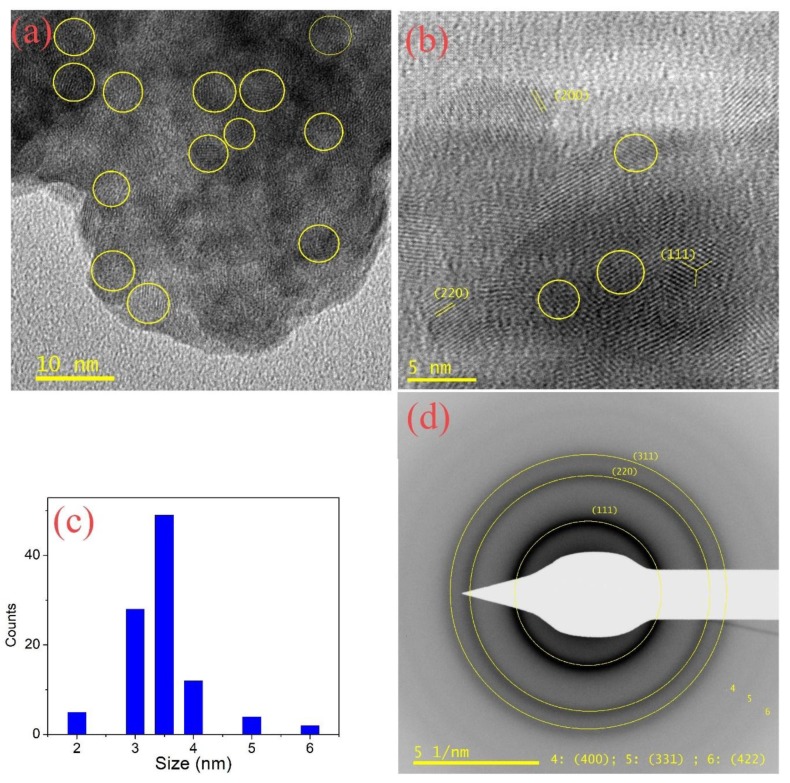
HRTEM images of the S10 sample: (**a**) HRTEM with a scale bar 10 nm; (**b**) HRTEM with a scale bar 5 nm. (**c**) Nanoparticles Size distribution. (**d**) Selected area electron diffraction (SAED).

HRTEM was used to obtain the size and morphological shape of the prepared quantum dots. Figure 4 exhibits a typical HRTEM image with different amplifications and a related selected area electron diffraction (SAED) pattern along with the size distribution of the ternary alloy sample S10. The quantum dots were nearly spherical in shape with an estimated average size of approximately 3.5 nm. The size of the quantum dots that was obtained from HRTEM was found to be slightly larger when compared to the one that was calculated from the XRD analysis. The major reason behind this result is that the X-ray only measures the size of the nanocrystalline core.

Figure 4d shows the SAED of the S10 sample; the appearance of clear diffraction rings is an indication of the good crystallinity of the sample. The lattice planes that appeared were indexed with (111), (220), (311), (400), (331), and (422) cubic planes with a smaller interplanar spacing d in comparison with the bulk CdSe. These results confirmed that the predominant phase for the samples had a modified cubic structure, which was compatible with the XRD results.

### 4.4. X-ray Photoelectron Spectroscopy (XPS)

**Figure 5:**
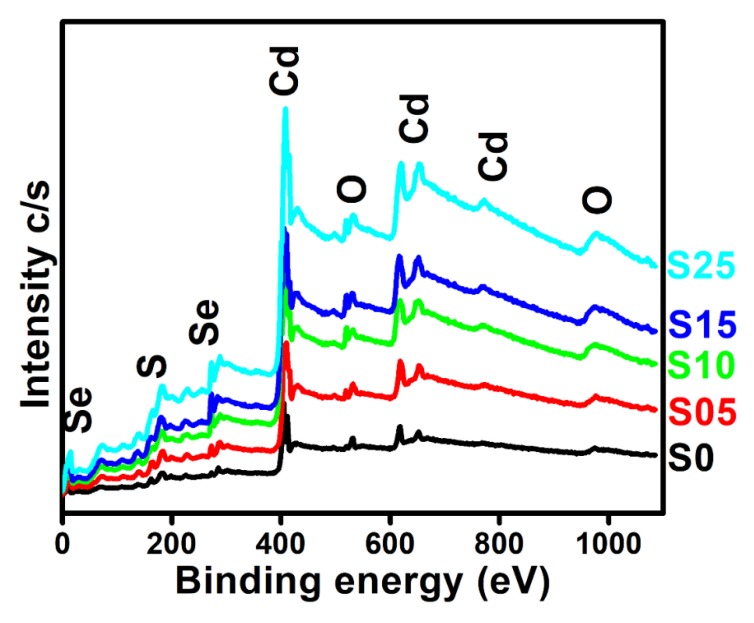
XPS survey spectra for binary CdSe and ternary CdSSe with different contents of sulfur.

The surface analysis, chemical composition, and the effect of sulfur doping of the prepared samples were investigated through XPS. The survey scan (Figure 5) showed the presence of Cd (3d), Se (3d), S (2p), and O (1s) elements with the different atomic percentages as detected by the elemental analysis.

**Figure 6:**
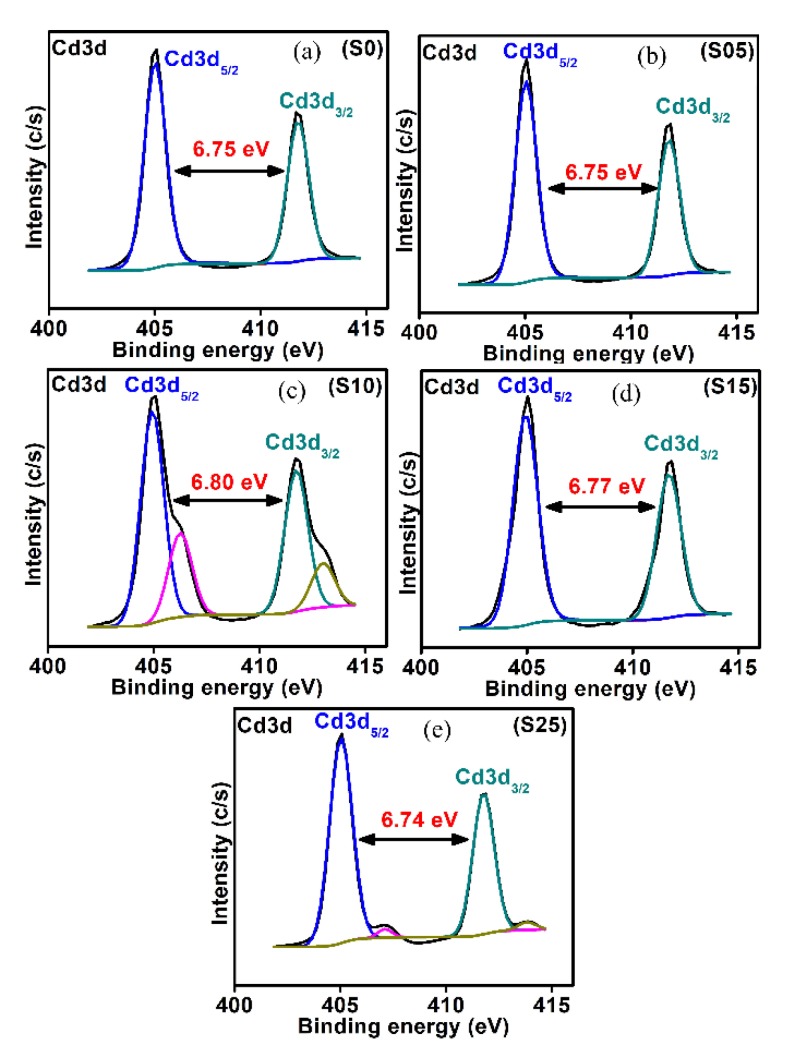
Chemical state analysis of Cd3d for the CdSe_1−*x*_S*_x_* nanostructures with different content of sulfur. (**a**) *x* = 0.0 (S0 sample); (**b**) *x* = 0.05 (S05 sample); (**c**) *x* = 0.1 (S10 sample); (**d**) *x* = 0.15 (S15 sample); (**e**) *x*= 0.25 (S25 sample).

The effect of sulfur contents on the chemical state of cadmium was investigated. The chemical state analysis of the Cd (3d) of CdSSe with different percentages of sulfur are shown in Figure 6. For the binary CdSe quantum dots, cadmium revealed two peaks around 405.02 ± 0.03 and 411.70 ± 0.09 eV, which were attributed to the 3d_5/2_ and 3d_3/2_ levels of cadmium in CdSe [53]. The separation energy between the 3d_5/2_ and 3d_3/2_ levels for the binary CdSe was found to be 6.75 eV. For the ternary structures of the samples with S contents of 10% and 15%, the separation energy between 3d_5/2_ and 3d_3/2_ levels of Cd slightly increased. The increase in the splitting of the two signals of Cd in the ternary alloy with 10% and 15% of S relative to the binary CdSe may have arisen from covering some of the Cd ions with sulfur. The variation in the peak positions (energy levels of 3d_5/2_ and 3d_3/2_) reflected the change in the crystalline structure by changing the composition of the nanostructure [54]. In addition to the change of the splitting of the 3d_5/2_ and 3d_3/2_ levels of cadmium, there was a variation in the atomic percentage of these two levels by the increasing S contents. Moreover, some broadening was noticed in the Cd structure, which had more adsorption of oxygen from the capping molecules. Additionally, other structures were observed for the samples S10 and S25 (Figure 6), which had been assigned to Cd at the surface coordinated with 3-MPA capping molecules or an oxidized cadmium surface. Previously published findings showed a similar structure for CdSe nanostructures dispersed in pyridine and attributed to oxidized Cd on the surface of the nanoparticles [55].

**Figure 7:**
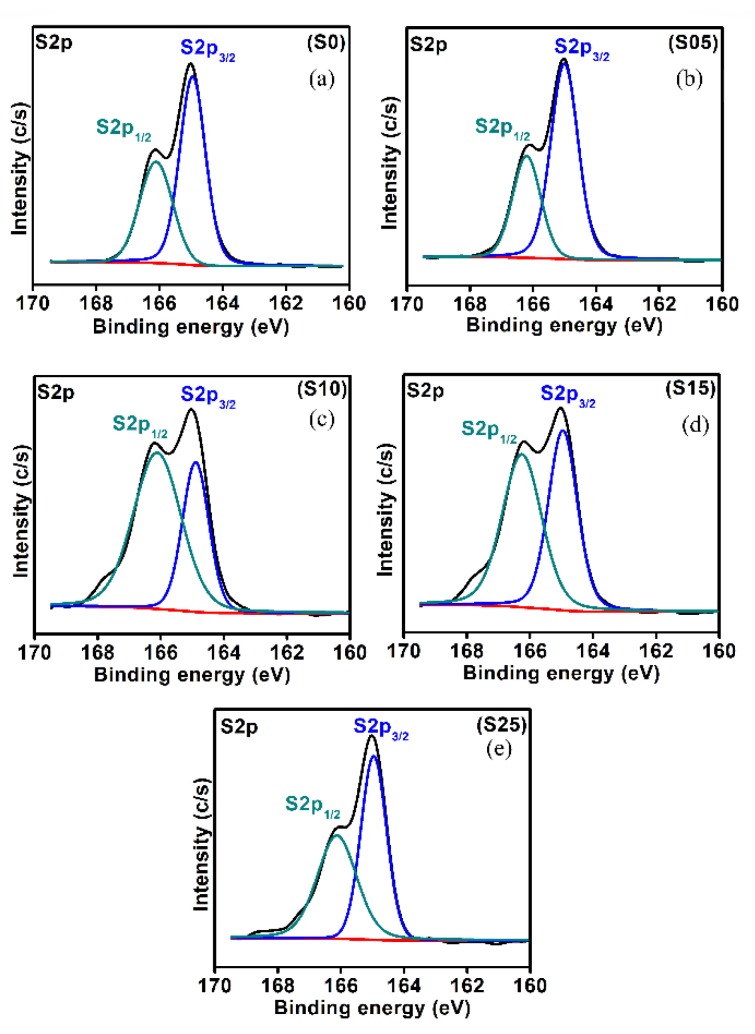
Chemical state analysis of S2p for the CdSe_1−*x*_S*_x_* nanostructures with different content of sulfur. (**a**) *x* = 0.0 (S0 sample); (**b**) *x* = 0.05 (S05 sample); (**c**) *x* = 0.1 (S10 sample); (**d**) *x* = 0.15 (S15 sample); (**e**) *x*= 0.25 (S25 sample).

The chemical states of sulfur for different nanostructures with various S contents were investigated. Figure 7 shows the chemical state analysis of S_2p_ for the CdSe and ternary CdSSe quantum dots. The S_2p_ exhibited two peaks around ~164.95 ± 0.05 and ~166.11 ± 0.02 eV, which were attributed to S_2p3/2_ and S_2p1/2_, respectively [53]. As shown in Figure 7, the atomic percentages of S_2p3/2_ and S_2p1/2_ were changed with the increasing S contents. The atomic percentages of S_2p1/2_ increased relative to S_2p3/2_ with increasing S content up to 15%, and decreased for the sample doped with 25%. The ratio of S_2p3/2_ was found to be 34.18 to 65.96%, whereas the variation in S_2p1/2_ was about 34.04 to 65.82%. The variation in S_2p3/2_ and S_2p1/2_ with the change in experimental condition proved the presence of sulfur from the doping and capping molecules on the surface of the prepared CdSe quantum dots.

### 4.5. UV-Vis Absorption

**Figure 8:**
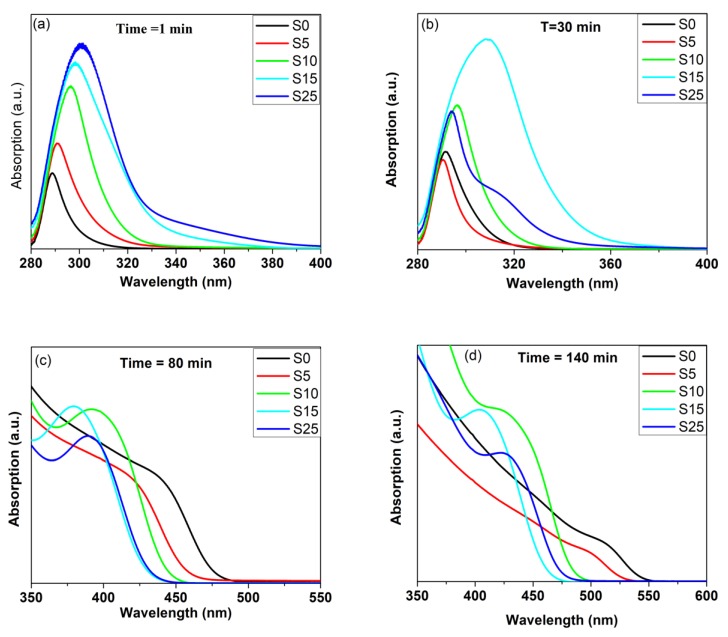
Optical absorption spectra of aliquots of binary CdSe and ternary alloy with different percentages of S: (**a**) After one minute of reaction, (**b**) after 30 min, (**c**) after 80 min, and (**d**) after 140 min.

**Figure 9:**
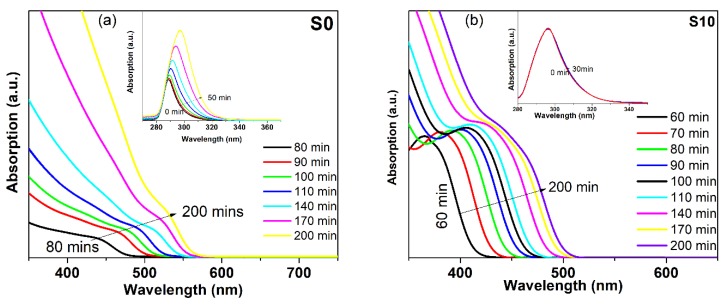
Optical absorption spectra during growth at different reaction times of: (**a**) binary CdSe (S0) and (**b**) ternary alloy with 10% of sulfur (S10).

**Figure 10:**
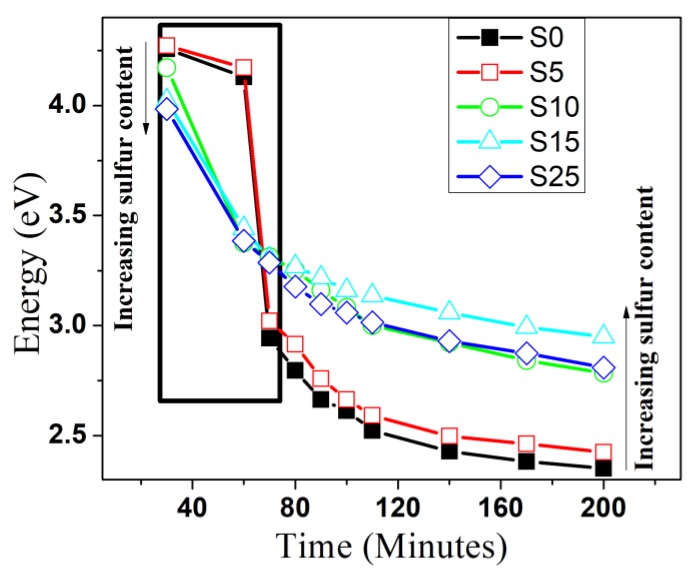
Effect of time on the position of the absorption maximum for the CdSe bare and alloyed structure with different S contents.

Substitutions of Se ions into the CdSe crystal lattice by sulfur anions is an additional method that gives another dimension to the engineering of the band gap. To produce CdSe_(1−*x*)_ S*_x_* nanocrystallites, a one-step route was executed by the concurrent reaction of Se^2−^ and S^2−^ with Cd^2+^ ions, followed by the addition of sodium borohydride as a reducing agent for the Se source. Figure 8a shows the optical absorption spectra of aliquots of binary CdSe and the ternary alloy with different percentages of S after one minute of injection of Se and S precursors and the addition of sodium borohydride. By adding sulfur, the absorption very clearly shifted to a lower energy with increasing S content and formed a long absorption tail. It is expected that the interaction of S with cadmium is different in reactivity and consequently in electronic properties. The Cd–S bonds are shorter than the Cd–Se bonds due to the smaller atomic radii of S. The electronegativity of S is higher than Se, so, the distribution of the charges of the S–Cd and Se–Cd bonds are different. These factors, along with the structures in initial growth, lead to the formation of many defects due to the presence of vacancies and interstitials in the nanostructures [56]. The presence of such defects leads to the formation of a long absorption tail as the S content is increased for this infant nanostructure. Figure 8b shows the alloyed structures along with the binary CdSe refluxed for 30 min. Obviously, the alloyed structures still shifted to a lower energy relative to the binary semiconductor except for the sample with 5% of S content, which shifted to a higher energy. In addition, the sample with 25% of S content revealed a pronounced shoulder and shifted to a higher energy relative to that one that had 15% of S. These results indicate that the ratio of chalcogenides ions had a pronounced effect on the growth of the alloyed structure. The optical absorption of the alloyed structures along with binary CdSe refluxed for 80 min and 140 min are shown in Figure 8c,d. Clearly, the behaviors were different and the absorption shifted to a higher energy with increasing S content of up to 15% sulfur. All spectra for the samples refluxed at 140 min shifted to a lower energy relative to the samples refluxed for 80 min due to the increase of nanocrystallite sizes. Additionally, the evolution of the optical properties of CdSe and CdSe_(1−*x*)_ S*_x_* alloyed QDs was investigated at different times after injecting the chalcogenide sources. Different molar ratios of the S/Se + S precursor were introduced to the preparation with the same reaction time of 200 min to synthesize the ternary alloy with different molar fractions of S. Figure 9a,b show the spectral evolution growth at different times of the S0 and S10 samples. With increasing time, the absorption spectrum shifted to a lower energy and a clear separated absorption band appeared after 70 min for binary CdSe. However, for the alloyed structure, the separated absorption band appeared at a shorter time in comparison with the bare CdSe. This behavior was attributed to the change in ionic strength of the reactive species for the two cases. Figure 10 shows the effect of time on the position of the absorption maximum for the CdSe bare and alloyed structure. Obviously, the behavior of growth was different for different compositions for the first 90 min of growth, while the growth kinetics after 90 min of the ternary alloy and binary nanocrystals were similar. Through the comparison of the energies of the absorption band of different compositions of the samples refluxed more than 90 min as shown clearly in Figure 10, the energy of the first absorption band increased with increasing S content up to 15% and decreased for the sample doped with 25%. This blue shift could be attributed to the tuning of the band gap of alloyed nanostructures from a wider band gap compound of CdS. In the alloyed nanostructures, the effective exciton mass should be modulated by varying the composition of the ternary CdSSe.

**Figure 11:**
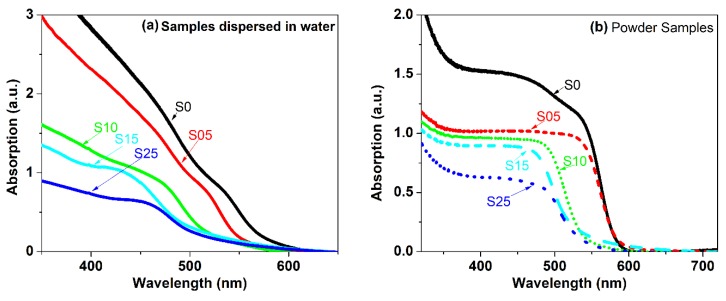
Absorption of CdSe_(1−*x*)_ S*_x_* with different concentrations of S of the precipitated samples (**a**) as a suspension in water (**b**) in a powder form.

**Figure 12:**
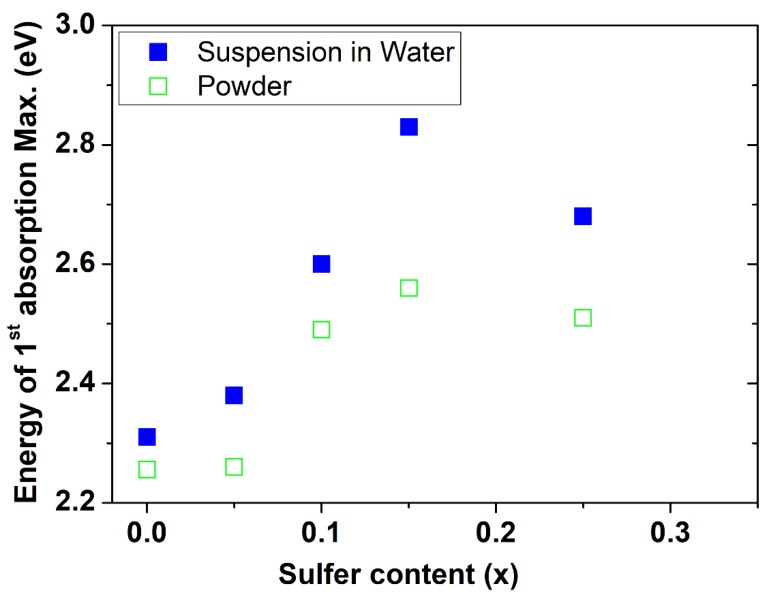
Comparison of the position of the energy of the first absorption maximum for the samples in powder form and dispersed in water.

The absorption of CdSe_(1−*x*)_S*_x_* with different concentrations of S of the precipitated samples was measured in powder form and as a suspension in water and revealed valuable information (Figure 11). The absorption onset and the maximum absorption were determined from the second derivative of the absorption spectra. Figure 12 shows the comparison of the position of the energy of the first maximum absorption for the samples in powder form and dispersed in water. The comparison of the absorption spectra of the nanopowder with the suspension of powder in water provided two important results: (i) the energy of the first absorption maximum and absorption onset of the nanopowder which were suspended in water shifted to a higher energy relative to the corresponding spectra of the powders, and (ii) the shift of the absorption maximum and absorption onset to a higher energy of the CdSSe alloy were larger than the shift of the bare CdSe. These results were attributed to the following reasons: (1) the differences between the dielectric constants of air and water (water = 1.78 and of air = 1), the change of the ratio of the dielectric constant of the environmental media leads to variation of electron-hole energy states and wave functions due to the change in the coulomb attraction potential and enhancement of the binding energy [57,58], and (2) the differences of the confinement barrier for the samples in powder form and suspended in water; a previous study showed that the decrease of the barrier matrix potential energy led to a decrease of the excitation energy [59]. The increase in the difference between the energy of the absorption maximum of the powder and the suspension of the alloy CdSSe in water in comparison with the bare CdSe reflects that the alloy structures had less barrier potential due to alloying with wide band gap material.

The sizes of the nanoparticles were calculated for the CdSe and CdSSe alloys according to [30]:

(4) $$E_{g}\left(x,d\right)\;=\;x\left[E_{g}\left(CdS,\infty \right)\;+\;\frac{a_{1}}{d}\;+\;\frac{c_{1}}{d^{2}}\right]\;+\;\left(1\;-\;x\right)\left[E_{g}\left(CdSe,\infty \right)\;+\;\frac{a_{2}}{d}\;+\;\frac{c_{2}}{d^{2}}\right]-\;b\left(d\right)x(1\;-\;x)$$

where Eg(*x*,d) is the band gap of the nanocrystallite; d is the nanocrystallite diameter; and Eg(CdS, ∞) and Eg(CdSe, ∞) are the bulk band gaps of CdS and CdSe, respectively. a_1_, a_2_, c_1_, and c_2_ are empirical fit parameters and have values of 8.4 (eV. A), 19.6 (eV. A), 245 (eV. A^2^), and –28.8 (eV. A^2^), respectively. For binary CdSe with *x* = 0 different values for a_2_ and c_2_ were applied to fit the experimental size (a_2_ = 20.1 eV. A and c_2_ = 29 eV. A^2^). The nanocrystallite sizes calculated with Equation (2) are listed in Table 1 and were in good agreement with the calculated sizes from the X-ray diffraction spectroscopy. The bowing parameter, applied for calculations of the sample doped with low concentrations (5%) of sulfur, was 0.2, which lies between the previously reported values for bulk materials of 0.3 and less than 0 [60,61].

With regard to the ternary alloy with more content of sulfur, the bowing constant was equal to 1 and increased with increasing sulfur content and decreasing size of the quantum dots. For quantum dots with a small size, the surface plays an important role in the nanostructure properties due to an increase of surface to volume ratio and consequently effects the bowing constant. The high values of the bowing constant suggest that the prepared ternary alloy was a compositionally graded nanostructure.

### 4.6. Luminescence

**Figure 13:**
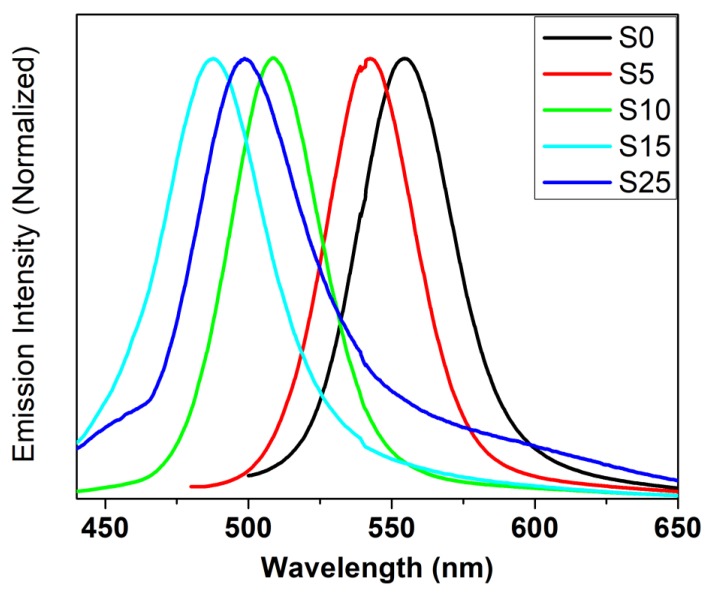
Normalized luminescence spectra of the CdSSe alloyed structure with different compositions.

Figure 13 shows the luminescence spectra of the CdSSe alloyed structure with different compositions. The luminescence emission of the binary and alloyed nanocrystallites revealed one emission band for all samples. Furthermore, the position of the emission band showed a blue shift with the increase in the S content. Exchange of the sulfide for selenide resulted in a smaller effective electron and hole masses pushing the system deeper into the Q-state regime. In addition, a small decrease in the nanocrystallite sizes of the ternary alloy was observed as revealed by X-ray analysis.

The position of the maxima of the emission band shifted by about 0.03 eV from the first absorption maximum, which we called Stock’s shift. This result indicates that the emission originated from the band to band recombination, which we called the near band edge emission. The smallest value of Stock’s shift was detected for the sample doped with sulfur at 10% (S10).

The effects of varying the content of sulfur on the full width at half maximum were investigated. The FWHM of the binary CdSe nanocrystallite was about 37 nm and decreased to 33 nm for the sample when doped with 10% of sulfur. An increase of nearly 3 to 6 nm was observed when the sample was doped with a relatively high concentration of S, which was attributed to intrinsic defects due to vacancies. The improvement in the emission of the sample doped with 10% may be explained by the increase of oxides on the surface by the coordination of carboxylate or native oxygen. The previous investigation through a combination of photoluminescence and Raman spectroscopy proved that the increase in the coordination of carboxylate on the surface of the quantum dots improved the band emission [52].

**Figure 14:**
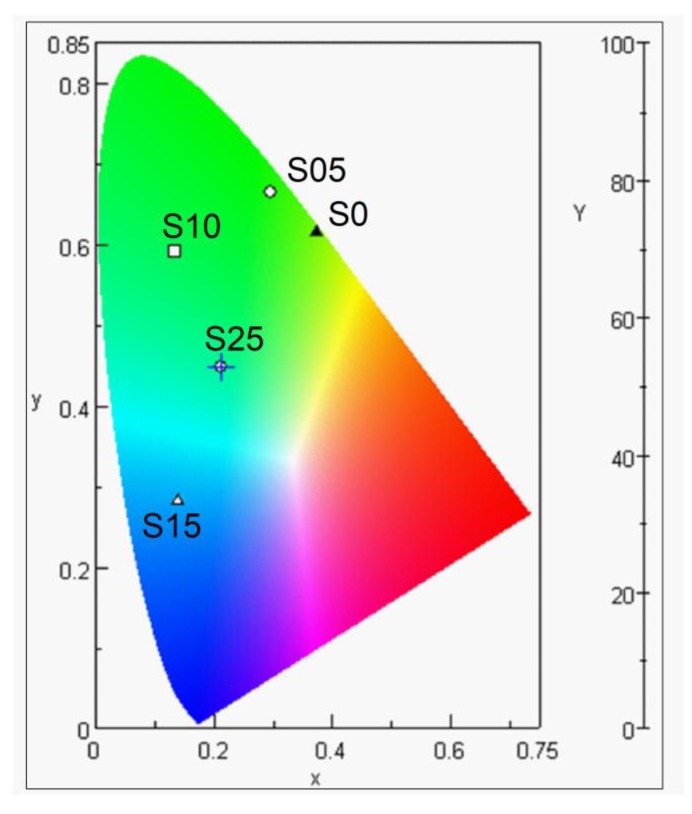
Chromaticity diagram for the emission color of the samples doped with different percentages of S.

The chromaticity diagram for the emission color of the samples doped with different percentages of S is shown in Figure 14. The chromaticity coordinates *x*, *y* and *z* were determined as follows:

(5) $$x\;=\;\frac{X}{X\;+\;Y\;+\;Z}$$

(6) $$y\;=\;\frac{Y}{X\;+\;Y\;+\;Z}$$

(7) $$z\;=\;\frac{Z}{X\;+\;Y\;+\;Z}$$

**Figure 15:**
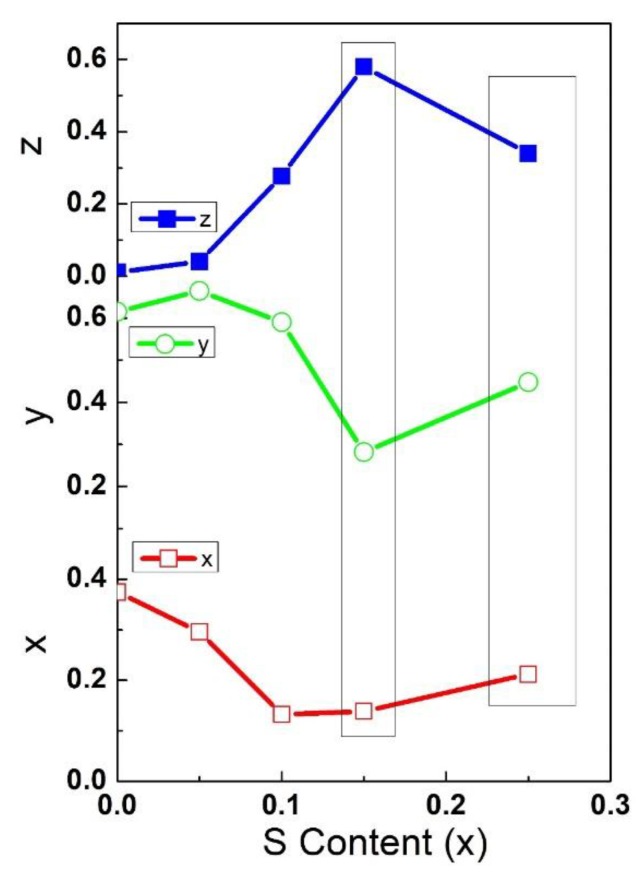
Variation of *x*, *y*, and *z* values against amount of S content.

The values of *x*, *y*, and *z* are directly related to the amount of specific color. The large value of x means a remarkable amount of red light and a large value of *y* indicates a large amount of green light, while a large value of *z* and the small amount of *x* and *y* indicates a large amount of blue light. According to the calculated values of the chromaticity coordinates, the relationship between *x*, *y*, and *z* were plotted against the amount of S content as shown in Figure 15. Clearly, with an increase in the S content by 15%, the *x* and *y* values showed a decreasing trend whereas the *z* value increased. The increase in the *z* value showed an increase in the energy of the emitted light and may have originated from the deeper quantum state that arises from the substitution of Se by S.

## 5. Conclusions

In the present study, the synthesis of water soluble CdS*_x_*Se_1−*x*_ alloy quantum dots by the one-pot method at low temperatures and capped 3-mercaptopropionic acid was reported. The quantum dots were prepared at low temperature with a new combination of precursors and without the need for a free oxygen atmosphere. Consequently, the prepared quantum dots are easy and cost-effective to utilize in various applications. The absorption maximum and band gap of the prepared quantum dots could be tuned in the visible region by changing the sulfur content. The dependence of the size and band gap were nonlinear with a small bowing constant of 0.2 for low concentrations, which is compatible with that of the bulk materials. Whilst for the sample prepared with a high content of sulfur, the bowing constant had a value of 1 and more than 1, which indicated the formation of graded diffusion alloy structures. The band edge emissions with a small full width at half maximum dominated for the alloyed structure with different S contents, which makes them suitable for biological labels and optoelectronic applications. The prepared alloyed quantum dot structures in this work showed tuning emissions that covered a wide range of visible light that originated from the band to band recombination and had a small FWHM. Consequently, these nanostructure alloys could be blended with polymers to act as an emission layer for hybrid light emitting devices.

## Figures and Tables

**Table 1:** Band gap and size of the quantum dots using different techniques for CdSe and CdSe doped with different S contents.

Sample	S/Se + S	S/Se + S	Band Gap	Diameter (nm)	Diameter (nm)	Bowing Parameter
	Precursors	EDX	(eV)	X-ray	UV-Vis	
S0	0	0.139	2.26	3.75	3.65	0
S05	0.05	0.183	2.26	3.80	3.80	0.2
S10	0.1	0.190	2.49	2.44	2.40	1
S15	0.15	0.196	2.56	2.10	2.13	1.3
S25	0.25	0.211	2.51	2.37	2.37	1.1

## References

[1] Rogach A.L. (2008). Semiconductor Nanocrystal Quantum Dots Synthesis, Assembly, Spectroscopy and Applications.

[2] Koole R., Groeneveld E., Vanmaekelbergh D., Meijerink A., Donegá C.D. (2014). Nanoparticles.

[3] Zheng H.M., Wu Y., Alivisatos A.P. (2009). Photovoltaic Devices Employing Ternary PbS*_x_*Se_1-*x*_ Nanocrystals. Nano Lett..

[4] Eck M., Krueger M. (2016). Correlation between CdSe QD Synthesis, Post-Synthetic Treatment, and BHJ Hybrid Solar Cell Performance. Nanomaterials.

[5] Mei X., Wu B., Guo X., Liu X., Rong Z., Liu S., Chen Y., Qin D., Xu W., Hou L. (2018). Efficient CdTe Nanocrystal/TiO_2_ Hetero-Junction Solar Cells with Open Circuit Voltage Breaking 0.8 V by Incorporating a Thin Layer of CdS Nanocrystal. Nanomaterials.

[6] Li Q., Li X., Wageh S., Al-Ghamdi A.A., Yu J. (2015). CdS/graphene nanocomposite photocatalysts. Adv. Energy Mater..

[7] Wang X., Li Y., Liu S., Zhang L. (2016). N-doped TiO_2_ Nanotubes as an Effective Additive to Improve the Catalytic Capability of Methanol Oxidation for Pt/Graphene Nanocomposites. Nanomaterials.

[8] Wageh S., Almazroai L.S., Alshahrie A., Al-Ghamdi A.A. (2018). Enhanced Visible Light Photo-Catalytic Activity of ZnO and Ag-Doped ZnO (ZnO:Ag) Nanoparticles. J. Nanosci. Nanotechnol..

[9] Prasad S., AlHesseny H.S., AlSalhi M.S., Devaraj D., Masilamai V. (2017). A High Power, Frequency Tunable Colloidal Quantum Dot (CdSe/ZnS) Laser. Nanomaterials.

[10] Vidor F.F., Meyers T., Hilleringmann U. (2016). Inverter Circuits Using ZnO Nanoparticle Based Thin-Film Transistors for Flexible Electronic Applications. Nanomaterials.

[11] Chan W.C., Nie S. (1998). Quantum dot bioconjugates for ultrsensitive nonisotopic detection. Science.

[12] Ruan C., Zhang Y., Lu M., Ji C., Sun C., Chen X., Chen H., Colvin V.L., Yu W.W. (2016). White Light-Emitting Diodes Based on AgInS_2_/ZnS Quantum Dots with Improved Bandwidth in Visible Light Communication. Nanomaterials.

[13] Wageh S. (2014). Light Emitting Devices Based on CdSe Nanoparticles Capped with Mercaptoacetic Acid. IEEE J. Quantum Electron..

[14] Rogach A.L., Harrison M.T., Kershaw S.V., Kornowski A., Burt M.G., Eychmuller A., Weller H. (2001). Colloidally Prepared CdHgTe and HgTe Quantum Dots with Strong Near-Infrared Luminescence. Phys. Status Solidi B.

[15] Gao F., Liu Y., Fan Y., Zhao D. (2014). Synthesis of N-acetyl-L-cysteine-capped ZnCdSe quantum dots via hydrothermal method and their characterization. Sci. Technol. Adv. Mater..

[16] Wageh S., Badr M.H. (2008). Cd_1-*x*_Zn*_x_*S Nanoparticles Stabilized by a Bifunctional Organic Molecule. Physica E.

[17] Al-Rasheedi A., Wageh S., Al-Zhrani E., Al-Ghamdi A. (2017). Structural and optical properties of CdZnTe quantum dots capped with a bifunctional Molecule. J. Mater. Sci. Mater. Electron..

[18] Ramírez-Herrera D.E., Rodríguez-Velázquez E., Alatorre-Meda M., Paraguay-Delgado F., Tirado-Guízar A., Pablo Taboada P., Pina-Luis G. (2018). NIR-Emitting Alloyed CdTeSe QDs and Organic Dye Assemblies: A Nontoxic, Stable, and Efficient FRET System. Nanomaterials.

[19] Baslak C., Arslan G., Kus M.Y. (2016). Removal of Rhodamine B from water by using CdTeSe quantum dot-cellulose membrane composites. RSC Adv..

[20] Gurusinghe N.P., Hewa-Kasakarage N.N., Zamkov M. (2008). Composition-tunable properties of CdS*_x_*Te_1-*x*_ alloy nanocrystals. J. Phys. Chem. C.

[21] Wageh S. (2016). Ternary ZnS:Te nanoparticles capped with 3-mercaptopropionic acid prepared in aqueous media. J. Mater. Sci. Mater. Electron..

[22] Hassanien A.S., Akl A.A. (2016). Effect of Se addition on optical and electrical properties of chalcogenide CdSSe thin films. Superlattices Microstruct..

[23] Hossain M.A., Jennings J.R., Mathews N., Wang Q. (2012). Band engineered ternary solid solution CdS_x_Se_1−__x_-sensitized mesoscopic TiO_2_ solar cells. Phys. Chem. Chem. Phys..

[24] Kang J.H., Myung Y., Choi J.W., Jang D.M., Lee C.W., Park J., Cha E.H. (2012). Nb_2_O_5_ nanowire photoanode sensitized by a composition-tuned CdS_x_Se_1−__x_ shell. J. Mater. Chem..

[25] Michalet X., Pinaud F.F., Bentolila L.A., Tsay J.M., Doose S., Li J.J., Sundaresan G., Wu A.M., Gambhir S.S., Weiss S. (2005). Quantum dots for live cells, in vivo imaging, and diagnostics. Science.

[26] Tang L., Zhang C.L., Song G.M., Jin X., Xu Z. (2013). In vivo skin penetration and metabolic path of quantum dots. Sci. China Life Sci..

[27] Elbaum R., Vega S., Hodes G. (2001). Preparation and surface structure of nanocrystalline cadmium sulfide (Sulfoselenide) precipitated from dimethyl sulfoxide solutions. Chem. Mater..

[28] Jang F., Jun S., Pu L. (2003). High quality CdSeS nanocrystals synthesized by facile single injection process and their electroluminescence. Chem. Commun..

[29] Qian H.F., Li L., Ren J.C. (2005). One-step and rapid synthesis of high quality alloyed quantum dots (CdSe-CdS) in aqueous phase by microwave irradiation with controllable temperature. Mater. Res. Bull..

[30] Swafford L.A., Weigand L.A., MJ Bowers M.J., McBride J.R., Rapaport J.L., Watt T.L., Dixit S.K., Feldman L.C., Rosenthal S.J. (2006). Homogeneously alloyed CdS_x_Se_1−__x_ nanocrystals: Synthesis, characterization, and composition/size-dependent band gap. J. Am. Chem. Soc..

[31] Ouyang J.Y., Vincent M., Kingston D., Descours P., Boivineau T., Zaman M.B., Wu X., Yu K. (2009). Noninjection, one pot synthesis of photoluminescent colloidal homogeneously alloyed CdSeS quantumdots. J. Phys. Chem. C.

[32] Zou Y., Li D.S., Yang D. (2010). Noninjection synthesis of CdS and alloyed CdS_x_Se_1−__x_ nanocrystals without nucleation initiators. Nanoscale Res. Lett..

[33] Unl¨u C., Tosun G.U., Sevim S., Özçelik S. (2013). Developing a facile method for highly luminescent colloidal CdS_x_Se_1−__x_ ternary nanoalloys. J. Mater. Chem. C.

[34] Charles Y., Cao Y.C., Wang J. (2004). One-Pot Synthesis of High-Quality Zinc-Blende CdS Nanocrystals. J. Am. Chem. Soc..

[35] Concina I., Natile M.M., Braga A., Vomiero A., Morandi V., Ortolani L., Ferroni M., Sberveglieri G. (2010). One pot synthesis of bi-linker stabilised CdSe quantum dots. J. Phys. Conf. Ser..

[36] Khanna P.K., Srinivasa Rao S., Patil K.R., Singh N., Mehta B.R. (2010). One-pot synthesis of oleic acid-capped cadmium chalcogenides (CdE: E *=* Se, Te) nano-crystals. J. Nanopart. Res..

[37] Ouyang J., Kuijper J., Brot S., Kingston D., Wu X., Leek D.M., Hu M.Z., Ripmeester J.A., Yu K. (2009). Photoluminescent Colloidal CdS Nanocrystals with High Quality via Noninjection One-Pot Synthesis in 1-Octadecene. J. Phys. Chem. C.

[38] Yu K., Ouyang J., Zaman M.B., Johnston D., Yan F.J., Li G., Ratcliffe C.I., Leek D.M., Wu X., Stupak J. (2009). Single-Sized CdSe Nanocrystals with Bandgap Photoemission via a Noninjection One-Pot Approach. J. Phys. Chem. C.

[39] Jia J., Tian J., Mi W., Tian W., Liu X., Dai J., Wang X. (2013). Growth kinetics of CdSe nanocrystals synthesized in liquid paraffin via one-pot method. J. Nanopart. Res..

[40] Chen X., Lou Y., Burda C. (2004). Spectroscopic investigation of II.VI core-shell nanoparticles: CdSe/CdS. Int. J. Nanotechnol..

[41] Mekis I., Talapin D.V., Kornowski A., Haase M., Weller H. (2003). One-Pot Synthesis of Highly Luminescent CdSe/CdS Core-Shell Nanocrystals via Organometallic and “Greener” Chemical Approaches. J. Phys. Chem. B.

[42] Niu Y., Pu C., Lai R., Meng R., Lin W., Qin H., Peng X. (2017). One-pot/three-step synthesis of zinc-blende CdSe/CdS core/shell nanocrystals with thick shells. Nano Res..

[43] Nguyen T.-L., Michael M., Mulvaney P. (2014). Synthesis of Highly Crystalline CdSe@ZnO Nanocrystals via Monolayer-by-Monolayer Epitaxial Shell Deposition. Chem. Mater..

[44] Lim J., Jun S., Jang E., Baik H., Kim H., Cho J. (2007). Preparation of Highly Luminescent Nanocrystals and Their Application to Light-Emitting Diodes. Adv. Mater..

[45] Zhan H., Zhou P., Pan K., He T., He X., Zhou C., He Y. (2013). One-pot aqueous-phase synthesis of ultra-small CdSe/CdS/CdZnS core–shell–shell quantum dots with high-luminescent efficiency and good stability. J. Nanopart. Res..

[46] AbouZeid K.M., Mohamed M.B., El-Shall M.S. (2016). Self-organization of Au–CdSe hybrid nanoflowers at different length scales via bi-functional diamine linkers. J. Nanopart. Res..

[47] Kong L., Chu X., Wang C., Yang X., Zhou L. (2017). Non-injection and one-pot approach to CdSe: Eu^3+^ hybrid nanocrystals with tunable photoluminescence from green to red. J. Nanopart. Res..

[48] Verma S.K., Verma R., Li N., Xiong D., Tian S., Xiang W., Zhang Z., Xie Y., Zhao X. (2016). Fabrication and band engineering of Cu-doped CdSe_0.6_Te_0.4_-alloyed quantum dots for solar cells. Sol. Energy Mater. Sol. Cells.

[49] Bhattacharyya S., Zitoun D., Gedanken A. (2008). One-Pot Synthesis and Characterization of Mn^2^^+^-Doped Wurtzite CdSe Nanocrystals Encapsulated with Carbon. J. Phys. Chem. C.

[50] Wang R., Calvignanello O., Ratcliffe C.I., Wu X., Leek D.M., Zaman M.B., Kingston D., Ripmeester J.A., Yu K. (2009). Homogeneously-Alloyed CdTeSe Single-Sized Nanocrystals with Bandgap Photoluminescence. J. Phys. Chem. C.

[51] Rogach A.L., Katsikas L., Kornowski A., Su D., Eychmüller A., Weller H. (1996). Synthesis and characterization of thiol-stabilized CdTe nanocrystals. Ber. Bunsenges. Phys. Chem..

[52] Wageh S. (2007). Raman and Photoluminescence Study of CdSe Nanoparticles Capped with a Bifunctional Molecule. Physica E.

[53] John F., William F., Peter E., Kenneth D. (1995). Handbook of X-ray Photoelectron Spectroscopy.

[54] Jilani A., Iqbal J., Rafique S., Abdel-wahab M.S., Jamil Y., Al-Ghamdi A.A. (2016). Morphological, optical and X-ray photoelectron chemical state shift investigations of ZnO thin films. Optik.

[55] Katari JE B., Colvin V.L., Alivisatos A.P. (1994). X-ray Photoelectron Spectroscopy of CdSe Nanocrystals with Applications to Studies of the Nanocrystal Surface. J. Phys. Chem..

[56] Wageh S., Al-Ghamdi1 A.A., Yakuphanoglu F. (2013). Band edge emission of ZnS nanoparticles prepared by excess of thiourea as a source of sulfur. J. Sol.-Gel. Sci. Technol..

[57] Bruss L.E. (1986). Zero-dimensional “excitons” in semiconductor clusters. IEEE J. Quantum Electron..

[58] Takagahara T. (1993). Effects of dielectric confinement and electron-hole exchange interaction on excitonic states in semiconductor quantum dots. Phys. Rev. B.

[59] Laheld UE H., Einevoll G.T. (1997). Excitons in CdSe quantum dots. Phys. Rev. B.

[60] Wei S.-H., Zhang S.B., Zunger A. (2000). First-principles calculation of band offsets, optical bowings, and defects in CdS, CdSe, CdTe, and their alloys. J. Appl. Phys..

[61] Mane R.S., Lokhande C.D. (1997). Studies on chemically deposited cadmium sulphoselenide (CdSSe) films. Thin Solid Films.

